# Leydig cell tumor in a patient with 49,XXXXY karyotype: a review of literature

**DOI:** 10.1186/s12958-015-0071-7

**Published:** 2015-07-10

**Authors:** Salwan Maqdasy, Laura Bogenmann, Marie Batisse-Lignier, Béatrice Roche, Fréderic Franck, Françoise Desbiez, Igor Tauveron

**Affiliations:** Service d’endocrinologie, diabétologie et maladies métaboliques, CHU Clermont-Ferrand, F-63003 Clermont-Ferrand, France; UMR CNRS 6293, INSERM U1103, Université Clermont-Auvergne, Génétique Reproduction et Développement, BP 10448, 63177 Aubiere, France; Service de Médecine Nucléaire, Centre Jean Perrin, 58 rue Montalembert, F-63011 Clermont-Ferrand, France; SIPATH Clermont-Ferrand, F-63003 Clermont-Ferrand, France

**Keywords:** Klinefelter, 49,XXXXY, *Fraccaro’s* syndrome, Leydig cell tumor, Leydigioma, Endocrine function

## Abstract

49,XXXXY pentasomy or Fraccaro’s syndrome is the most severe variant of Klinefelter’s syndrome (KS) affecting about 1/85000 male births. The classical presentation is the triad: mental retardation, hypergonadotropic hypogonadism and radio ulnar synostosis. Indeed, the reproductive function of Fraccaro’s syndrome is distinguished from KS. Besides, Leydig cell tumors are described in cases of KS, but never documented in the Klinefelter variants.

We describe a young adult of 22 years old who presented with hyper gonadotropic hypogonadism, delayed puberty and bilateral micro-cryptorchidism. Chromosomal pentasomy was confirmed since infancy. Bilateral orchidectomy revealed a unilateral well-circumscribed Leydig cell tumor associated with bilateral Leydig cell hyperplasia.

Inspired from reporting the first case of Leydig cell tumor in a 49,XXXXY patient, we summarize the particularities of testicular function in 49,XXXXY from one side, and the risk and mechanisms of Leydig cell tumorigenesis in Klinefelter variants on the other side. The histological destructions in 49,XXXXY testes and hypogonadism are more profound than in Klinefelter patients, with early Sertoli, Leydig and germ cell destruction. Furthermore, the risk of Leydigioma development in KS and its variants remains a dilemma. We believe that the risk of Leydigioma is much higher in KS than the general population. By contrast, the risk could be lower in the Klinefelter variants with more than 3 supplementary X chromosomes, owing to an earlier and more profound destruction of Leydig cells rendering them irresponsive to chronic Luteinizing hormone (LH) stimulation.

## Background

Klinefelter syndrome (47,XXY or KS) is the commonest aneuploidy. It affects 1/650 male births (0.2 % of general population). Besides, more severe, fortunately rare, aneuploidies are also described; these include: 48,XXXY, 48,XXYY and 49,XXXXY. 48,XXYY affects 1/8000-1/40,000 male deliveries, while 48,XXXY affects 1/50,000. Pentasomy 49,XXXXY incidence is around 1/85000 male births [1, 2]. A 49,XXXXY karyotype is thought to arise from maternal non-disjunction during both stages of meiosis, retaining all the X chromosomes within the oocyte. The major endocrine issues of aneuploidies are hyper gonadotropic hypogonadism, testicular degenerative changes and the risk of testicular tumorigenesis. Indeed, Leydig cell tumors or Leydigioma are occasionally described in cases of KS, but never in the Klinefelter variants.

The objectives of this article are: we document herein, the first case of Leydig cell tumor associated with bilateral Leydig cell hyperplasia in a 49,XXXXY patient. Furthermore, we will review the particularities of testicular function in 49,XXXXY from one side and the mechanisms and Leydig cell tumorigenesis in Klinefelter variants on the other side.

## Case presentation

A young patient born in 1990 is followed up in our department for hypogonadism. At birth, facial dysmorphism characterized by microcephaly, hypertelorism, inclination of the palpebral fissures, small broad-based nose, micro retrognathia and a clinodactyly of the 5th finger were noticed immediately. A parasternal systolic murmur of left-to-right shunt was audible. The karyotype of the patient revealed 49,XXXXY aneuploidy. During his childhood, mental retardation and delayed milestones were documented with walking at age of 3.5 years, pronouncing words of two syllables at age of 21 months. At age of 15 years, he was described as a joyful teenager, who showed a substantial anxiety for unusual situations. His language was limited to about ten words. He was able to write his name correctly, to copy a short text and to count up to ten.

Clinical examination revealed a bilateral testicular ectopia and hypoplasia of the external genitalia. Musculoskeletal examination revealed scoliosis and bilateral radio ulnar synostosis. Moderate hypotonia was also noticed since his childhood, along with a significant fatigability.

At age of 13 years, puberty was absent (*Tanner* stage P1) and hyper gonadotropic hypogonadism was confirmed. The hormonal profile was in favour of early severe testicular failure (FSH 27 UI/L, LH 17 UI/L with undetectable testosterone levels). Testosterone therapy (50 mg/3 weeks, increased progressively to 125 mg/3 weeks) was initiated at the age of 15 years. Skeletal age was 12.5 years. Testosterone levels were consequently normalized to 5.4 ng/ml under the substitutive treatment, with FSH reduced to 9.2 UI/L and LH levels to 5.1 UI/L (normal levels: FSH 2.2-9.8 UI/L; LH 1.8-7 UI/L).

**Fig. 1:**
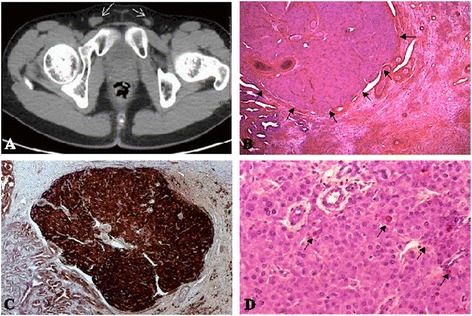
Identification of a Leydigioma in a patient with 49,XXXXY karyotype with bilateral testicular ectopia. **a** Identification of 1 and 1.5 cm diameter testes within the inguinal groin by computed tomography in adolescence. **b** Identification of a well circumscribed 2 mm diameter tumor in the testis. Tumor cells are hexagonal, with round uniform prominent nuclei. The cytoplasm is eosinophilic, or slightly pale due to lipid accumulation. Lipofuschine pigment is identified in steroid producing tumors. Some calcification and hyalinization of the stroma could be identified. Reinke crystals, pathognomonic for Leydigioma are present in only 40 % of the cases. They were absent in this case. **c** Immuno staining of the tumor by Calretinin, a specific marker of stroma cell tumors. **d** Ki67 immuno staining. the proliferative index is low in the benign tumors

Young adult, he measures 1.83 m and weighs 55 kg with a slender silhouette. His testicles were impalpable. An abdmino-pelvic tomography revealed both testicles in the inguinal position (Fig. 1a).

Bilateral orchidectomy was realized in 2012. The macroscopic examination reported right and left small testicles (1.5x1 cm). Excised tissues were fixed using 4 % paraformaldehyde (Sigma-Aldrich) and embedded in paraffin. Microscopic examination after Hematoxylin/Eosin staining (Sigma-Aldrich) on 5 μm tissue sections revealed that fibrous involution replaced the seminiferous tubules along with disappearance of Sertoli cells, associated with Leydig cell hyperplasia. No germ cells were detected. A well-circumscribed right-sided Leydig cell tumor was detected. The tumor was composed of hexagonal eosinophilic cells. Lipofusceine grains were observed without Reinke crystalloids. The nuclei were rounded with small nucleoli (Fig. 1b). Calretinin immuno staining (790-4467/Ventana Clone SP65) confirmed the nature of the sex cord stromal tumor (Fig. 1c). The appearance was monomorphic, without any significant proliferative activity (less than 1 %) (Fig. 1d). The proliferative activity was evaluated by immuno staining with human anti-Ki67 antibody (Ki67: 790-4286/Ventana Clone 30–9). Immunohistochemistry for calretinin and Ki-67 was conducted according to the manufacturer’s recommendations; the slides were then counterstained with haematoxylin.

Two years post-orchidectomy computed scanner revealed no metastases. Testosterone replacement therapy permitted to virilise the external genitalia (*Tanner* stage P3).

## Discussion

1. General

Harry Klinefelter published a report on nine men with testicular dysgenesis, elevated gonadotropins, micro orchidism, azoospermia, and gynecomastia in 1942 [3]. The hallmark of KS is hyper gonadotropic hypogonadism with testicular atrophy [4]. The diagnosis is usually evoked in infants with delayed milestones [5], and in male adults with sterility [6, 7].

49,XXXXY is the most severe form, accounting for 1.4-1.7 % of aneuploidies [2, 8, 9]. Described firstly in 1960 by *Fraccaro*, since then, we detected 176 cases in the literature. Some authors distinguish specific characteristics of this syndrome [1, 10, 11]. The classical presentation is the triad: mental retardation, hypogonadism and radio ulnar synostosis [2, 10–12]. Hypogonadism is severe and frequently associated with genital anomalies, which are characterized by micro penis, small testicles and scrotum, cryptorchidism and genital ambiguity. Gynecomastia is rare [10].

Some paradoxical features seem to be specific to 49,XXXXY; 49,XXXXY are usually shorter than the general population [13, 14]. Microcephaly is found in some children with 20 % reduction in cerebral volume [15], which is more severe than Klinefelter patients [16]. Focal neurological signs are possible, which are related to demyelination [15]. Language acquisition is late, and usually other alternatives of communication are used in adult age [17]. We analysed 176 published cases of 49,XXXXY in the literature. We noticed two principal malformations in the published cases: cardiac (22 patients, mostly patent ductus arteriosus) and urogenital (7 patients). The global mortality in aneuploidies in general and in 49,XXXXY in particular, studied in details in 3518 patients of a British cohort included between 1958 and 2003 (48 cases of 49,XXXXY), was elevated (SMR: 1.5-2) with congenital heart diseases, epilepsy and pulmonary embolism as major causes of mortality [8].

Indeed, our patient presented the classical triad of 49,XXXXY syndrome, which was associated with facial dysmorphism, psychological troubles, mental retardation and cardiac malformation. But, he had eunuchoid morphology as in Klinefelter patients, which is rare for 49,XXXXY.

2. Testicular function in 49,XXXXY

Indeed, the testicular anomalies are the hallmark of aneuploidies, starting from birth and on-going to adulthood [18]. Histological destructions in 49,XXXXY testes are more profound than Klinefelter patients. In contrast to KS, micro orchidism occurs in almost all cases of 48,XXYY, 48,XXXY and 49,XXXXY, with adult testicular volumes typically around 1–4 mL [14, 17].

In early adulthood, Klinefelter patients seem to enter normally in the puberty, with incomplete development of secondary sexual characters, but this is rare in the variants which are characterized by absence of puberty [19]. The endocrine profile of KS and the variants is characterized by normal levels of gonadotropins, AMH and Inhibin B with variable levels of testosterone in infancy [20–22]. In infants, Leydig cells are normally sensitive to the proliferative effect of LH but Sertoli cell sensitivity is questionable, post pubertal Sertoli cell resistance to FSH is definite [23]. Hypogonadism is more profound in the variants than the classical 47,XXY syndrome. This is due to a more profound testicular damage, associated with lower testosterone levels, even in infancy leading to ambiguous genitalia. The endocrine profile of aneuploidies is summarized in Table 1.

**Table 1:** Chronological evolution of clinical, histological and hormonal parameters of Klinefelter variants. Eunuchoid morphology and gynecomastia are absent in 49,XXXXY karyotype

Parameter	Infancy	Early puberty (12 years)	MidPuberty Tanner	Puberty Tanner
			II-III	III-IV
FSH	N	N	++	++++
LH	N	N	+	+++
T	-	N or -	+	- - -
E2	N	++	++	++
Inhibin B	N	N	- -	- - - -
AMH	N	N	- -	- - -
INSL3	N	N	- -	- - - -
Germ cells	Degeneration begun	Progressive degeneration	Accelerated degeneration in early puberty	- - - -
			Presence of spermatogonia only	
Clinical	Cryptorchidism		Subnormal Testis weight	Eunuchoid (+/−)
				Gynecomastia (+/−)
				Hypogonadism
				Testis atrophy

+, ++, +++, ++++: Mild, moderate, high, very high increase; −, − −, − − −: mild, moderate, severe decrease; − − − −: undetectable; N: Normal; FSH: Follicle stimulating hormone; LH: Luteinizing Hormone; T: Testosterone; E2: 17β oestradiol, AMH: Anti-Müllarian hormone; INSL3: Insulin Like 3

In our case, the hypogonadism was profound with no initiation of puberty. Gynecomastia was absent indicating no any testosterone secretion to be aromatized. Testicular ectopia was moderate but associated with a complete testicular fibrosis, with no germ or Sertoli cells identified. Therefore, we suggest that the chromosomal anomaly is responsible for early Sertoli and germ cell deterioration. Indeed, we suppose that the degree of damage of Sertoli cells, their sensitivity to follicle stimulating hormone (FSH) and germ cell destruction in patients with more than 3 X chromosomes is more profound. This is supported by the evidence that the chance of fertility or successful retrieval of gametes is limited to Klinefelter patients especially in mosaic forms than other aneuploidies. On the other side, Leydig cell deterioration is tardive. This is supported by the testicular decent even if incomplete (dependent on Insulin like 3 and testosterone secreted from foetal Leydig cells), Leydig cell hyperplasia (responsiveness to chronic Luteinizing hormone stimulation from puberty) and the presence of steroidogenesis in early puberty. Nevertheless, In comparison to 47, XXY, Leydig cell deterioration seems to be more profound and earlier in 49,XXXXY patients, as steroid synthesis is negligible manifested by higher risk of ambiguous genitalia and absent puberty.

3. Risk of Leydig cell tumors or Leydigioma

Testicular tumors are relatively rare, corresponding to 1 % of all human cancers [24]. Germ cell tumors and sex cord stromal tumors constitute the two predominant types. Since 1960s, the incidence of testicular cancer doubled in most of western countries [25]. Fortunately, only 20 % of these tumors appear during infancy (5–10 years old) [26]. Leydig cell tumors or Leydigioma represent 0.8–3 % of all testicular tumors in adulthood and 4-9 % of childhood testicular tumors [27–29]. Most of these tumors are benign, especially in infancy [30].

Testicular cancers are clearly linked to undescended testis, testicular dysgenesis syndrome (TDS) and infertility [31–38]. Malignancy occurs in 3.5–14.5 % of undescended testis. Thus, a higher risk of testicular cancers in Klinefelter patients and the other variants has been supposed. Leydig cell tumors and/or hyperplasia are not uncommon findings during the histological examination of human testicular biopsies, especially in patients with testicular atrophy, cryptorchidism, KS and androgen insensitivity syndrome [39, 40]. Indeed, a Danish Study published in 1995 (696 patients) and a British cohort of 3518 patients with Klinefelter variants including 48 patients with 49,XXXX, seem to be the most significant publications to explore the tumor risk. They showed a higher relative risk of mortality (RR = 1.5), but cancer related mortality was not different from the general population [9, 41]. More specifically, the risk of testicular cancers seemed to be equivocal to the general population in these studies. Nevertheless, extra gonadal (mostly mediastinal) germ cell tumors were more commonly reported in patients with KS in comparison to the general population, with a prevalence of 1 % [41–44]. Furthermore, in another study, Ahmad *et al.* measured the volume of Leydig cells histometrically on biopsies of 50 cases of KS; the mean volume was within normal limits [45]. Meanwhile, it is important to mention that the first two studies interested more specifically in cancer risk and did not take in consideration the benign tumors or hyperplasia and they were based on the data registries. On the other hand, Ahmad *et al.* analysis was based on retrospective data. By contrast, a recent Italian study screened the testes of 40 Klinefelter patients by ultrasound, magnetic resonance imaging and tumor markers. Over 3 years of follow up, 30 % of patients presented with either cysts or nodules, which were below 1 cm. But, no clinical, biological and radiological arguments for testicular cancer were identified. Moreover, no biological/morphological differences between those with or without a history of cryptorchidism were noticed [46]. More specifically, a French cohort was based on ultrasound screening the testes of 141 KS patients. 158 testicular nodules in 56 (40 %) patients were identified. 20 % of them had bilateral nodules. Indeed, only 12 patients (7.6 %) were operated and all of them suffered from Leydig cell hyperplasia and/or Leydig cell tumors.

On the other side, the karyotype anomalies in testicular tumor context are not well recognized; unfortunately, most studies interested in the testicular tumors neglected the karyotype of these patients [37, 47]. A French study of 45 tumors in infertile patients with 11 Leydig cell hyperplasia and 17 benign Leydig cell tumors, revealed that KS was found in 10 patients. Indeed, 12 Leydig cell tumors were identified in these ten patients and fortunately all were benign [48].

We analysed the literature for the published cases of testicular tumors in aneuploidies, over nearly 4000 patients with KS and its variants, we identified only 34 patients with gonadal and extra gonadal tumors. 20 patients had Leydig cell tumors, fortunately only two were malignant; teratomas were the second most common tumors described (Table 2).

**Table 2:** Reported cases of testis-related tumours in aneuploidies

References	No. of Patients	Karyotype	Tumour type
*Yoshida* [89]	One	47,XXY	Germ cell tumour
*Carroll* [90]	One	47,XXY	Germ cell tumour
*Sasagawa* [91]	Two	47,XXY	Germ cell tumours
*Baniel* [92]	One	47,XXY	Benign epidermal cyst
*Reddy* [93]	One	48,XXYY	Seminoma
*Isurugi* [94]	One	47,XXY	Seminoma
*Tada* [95]	One	47,XXY	Teratoma
*Stevens* [96]	One	47,XXY	Bilateral teratoma
*Matsuki* [97]	One	46,XX/47,XXY	Mature Teratoma
*Simpson* [98]	Two brothers	47,XXY	Teratomas
*Gustavson* [99]	Two	47,XXY	Bilateral Teratomas
*Ekerhovd* [100]	One	47,XXY	Sertoli cell tumour
*Lardennois* [101]	One	47,XXY	Bilateral Leydig cell tumours
*Soria* [102]	One	47,XXY	Malignant Leydig cell tumour
*Arduino* [103]	One	47,XXY	Benign Leydig cell tumour
*Dodge* [104]	One	47,XXY	Benign Leydig cell tumour
*Knyrim* [105]	One	47,XXY	Malignant Leydig cell tumour
*Poster* [106]	One	47,XXY	Benign Leydig cell tumour
*Okada* [107]	One	47,XXY	Benign Leydig cell tumour
*Westlander* [108]	One	47,XXY	Leydig cell tumour
*Heer* [109]	One	47,XXY	Leydig cell tumour
*Fishman* [110]	One	47,XXY	Benign Leydig cell tumour
*Miazlin* [77]	One	47,XXY	Benign Leydig cell tumour
*De Miguel* [111]	Five	47,XXY	Leydig cell hyperplasia in all

Returning to our patient, this is the first case of benign Leydigioma associated with bilateral Leydig cell hyperplasia in a Klinefelter variant. It is important to say that the literature today shows an equivocal or even a lower risk of testicular tumors in KS in comparison to the general population. Nevertheless, Leydigioma incidence is possibly underestimated, as early preventive orchidectomy is usually practiced; furthermore, no systemic screening is consensual to detect such tumors. As we reviewed above, 30 to 40 % of KS patients have testicular masses on imaging. To-date the real incidence of Leydig cell tumor in KS and its variants is difficult to be estimated; we believe that the risk is much higher than the general population. By contrast, the risk could be lower in the Klinefelter variants with more than 3 supplementary X chromosomes, owing to an earlier and more profound destruction of Leydig cells rendering it irresponsive to chronic Luteinizing hormone (LH) stimulation. That’s why, no Leydig cell tumor is yet reported in the literature (176 cases of 49,XXXXY).

4. Physiopathology of Leydig cell tumorigenesis

Undescended or ectopic testes are common in aneuploidies (about 14-28 % of the cases, 6 times higher than the male general population) [49, 50]. The peak age and histological distribution of tumor in undescended testis are similar to the scrotal testes. Most of the studies reported germ cell tumors (more than 90 % of the tumors) and rarely, Leydig cell tumors were described. Indeed, the mechanisms are multiple and remain unclear. One hypothesis is that cryptorchidism is not merely an incomplete descent of the testis, but it reflects a generalized defect in embryogenesis and results in bilateral dysgenetic gonads. The most compelling is that the risk of testicular carcinoma is not limited to the undescended testis, but it extends to the contralateral testis, even if it is normally descended. Thus, the increased risk of carcinoma cannot be attributed only to the local environmental factors, such as increased temperature in the abdomen versus the scrotum [51, 52].

Epidemiological studies identified common risk factors between infertility and testicular cancer. Hyper estrogenic and hypo androgenic status are the most commonly accepted risk factors for testicular cancer and infertility. Although cryptorchidism is a known risk of testicular cancer and infertility, the risk of testicular cancer among infertile men exceeds the frequency of cryptorchidism in the same population [37].

Many mechanisms and animal models explain how a Leydigioma could develop, nevertheless, the causes remain heterogeneous. Although LH plays an important role in Leydig cell proliferation, Leydig cell maturation and proliferation is affected by many other paracrine and endocrine signals, including anti müllerian hormone, inhibin, and other growth factors. The classical pathway was suggested since 1980 with chronic LH stimulation which induced Leydig cell hyperplasia/adenoma in rats [53]. Furthermore, anti-androgen therapy or androgen insensitivity syndrome inducing LH secretion resulted in the same phenotype [40, 54]. This hypothesis was confirmed with the description of activating mutations of LH receptors. Indeed, LH receptor is a trans membrane G protein coupled receptor, expressed on Leydig cells. Activating mutations of this receptor are reported and induce precocious puberty [55]. The chronic and permanent hyper activation of this receptor leads to inappropriate stimulation of cAMP pathway and Leydig cell hyperplasia. This mutation is usually found in children who present with Leydig cell tumor, and frequently responsible for precocious puberty [56–65]. Actually, Asp578His mutation was identified in more than 50 % of children with Leydig cell tumors (13/24 patients) (reviewed in [57]).

Besides, mice models of KS with 41,XXY karyotype [66], are characterized by small testes with Leydig cell hyperplasia [67]. This supports the role of chronic and permanent LH stimulation in the physiopathology of Leydig cell tumor [67, 68].

Leydig cell tumor/hyperplasia were also linked to many other situations than aneuploidies; these include: McCune-Albright syndrome, Carney complex, fumarate hydratase and cyclin dependent kinase (CDK) mutations. The first two syndromes are multi tumor syndromes affecting the endocrine system, including Leydig cells. Their physiopathology is near to that of Klinefelter variants with the activation of LH pathway (Activating mutations of α subunit of protein G (GNAS) for McCune-Albright syndrome [69–72] and hyperactivity of protein kinase A in Carney complex [73, 74]).

5. Management of Leydig cell tumors

Orchidectomy is indicated in cases of benign Leydig cell tumors (single, unilateral, well circumscribed tumor without hyper vascularization, necrosis, lithiasis or calcification on ultrasound) [75–77]. Owing to the favourable course of such usually small-sized tumors, some published studies advocated conservative or testis-sparing surgery [78–82]. Such option does not seem to be associated with an increased risk of recurrence. Conservative surgery seems to be important for young patients with single testicle with paternity desire [83, 84]. Frozen sections largely help to decide a more radical surgery or not [79, 82, 85]. The first 2 years of the diagnosis of malignant Leydig cell tumor are the most crucial for the prognosis of the patients, however, metastases up to 17 years post operation were also observed [86]. More radical surgery with retroperitoneal ganglion removal, chemotherapy, and/or radiotherapy could be used [30, 87, 88].

The dilemma of surgical choice is less advocated in adult patients with KS associated with undescended testis, advanced testicular failure or non-existence of fertility challenge. These concerns are usually present in Klinefelter variants. Thus, no benefits are awaited from testis-sparing surgical procedures.

## Conclusion

We present a case of 49,XXXXY sex polysomy, who shares a number of characteristics of the other 176 patients cases described in the literature, namely, hypogonadism with cryptorchidism, facial dysmorphism, musculoskeletal and cardiac malformations, and mental retardation seriously affecting language skills. He presented with a benign Leydig cell tumor. To our best knowledge, this is the first case of testicular Leydig cell tumor described in a patient affected by *Fraccaro* syndrome.

Even if it shares some characteristics with KS, 49,XXXXY syndrome has to be distinguished and be considered as the most severe form. Hypogonadism is severe, together with genital ambiguity and absent puberty evoking an earlier and more profound Sertoli, Leydig and germ cell destruction.

The risk of Leydig cell tumors in aneuploidies remains a dogma; it seems to be similar to the general population. Nevertheless, this incidence is possibly underestimated, as early preventive orchidectomy is usually practiced; furthermore, no systemic screening is consensual to detect such tumors. On the other side, karyotype study in patients with Leydig cell tumor/hyperplasia is seldom realized and needs to be more systematic. Finally, the most agreed physiopathology is related to chronic LH stimulation of Leydig cells.
